# Forseti: a mechanistic and predictive model of the splicing status of scRNA-seq reads

**DOI:** 10.1093/bioinformatics/btae207

**Published:** 2024-06-28

**Authors:** Dongze He, Yuan Gao, Spencer Skylar Chan, Natalia Quintana-Parrilla, Rob Patro

**Affiliations:** Center for Bioinformatics and Computational Biology, University of Maryland, College Park, MD 20742, United States; Program in Computational Biology, Bioinformatics and Genomices, University of Maryland, College Park, MD 20742, United States; Center for Bioinformatics and Computational Biology, University of Maryland, College Park, MD 20742, United States; Program in Computational Biology, Bioinformatics and Genomices, University of Maryland, College Park, MD 20742, United States; Department of Computer Science, University of Maryland, College Park, MD 20742, United States; Department of Biology, University of Puerto Rico, Mayagüez Campus, Mayagüez 00682, Puerto Rico; Center for Bioinformatics and Computational Biology, University of Maryland, College Park, MD 20742, United States; Department of Computer Science, University of Maryland, College Park, MD 20742, United States

## Abstract

**Motivation:**

Short-read single-cell RNA-sequencing (scRNA-seq) has been used to study cellular heterogeneity, cellular fate, and transcriptional dynamics. Modeling splicing dynamics in scRNA-seq data is challenging, with inherent difficulty in even the seemingly straightforward task of elucidating the splicing status of the molecules from which sequenced fragments are drawn. This difficulty arises, in part, from the limited read length and positional biases, which substantially reduce the specificity of the sequenced fragments. As a result, the splicing status of many reads in scRNA-seq is ambiguous because of a lack of definitive evidence. We are therefore in need of methods that can recover the splicing status of ambiguous reads which, in turn, can lead to more accuracy and confidence in downstream analyses.

**Results:**

We develop Forseti, a predictive model to probabilistically assign a splicing status to scRNA-seq reads. Our model has two key components. First, we train a binding affinity model to assign a probability that a given transcriptomic site is used in fragment generation. Second, we fit a robust fragment length distribution model that generalizes well across datasets deriving from different species and tissue types. Forseti combines these two trained models to predict the splicing status of the molecule of origin of reads by scoring putative fragments that associate each alignment of sequenced reads with proximate potential priming sites. Using both simulated and experimental data, we show that our model can precisely predict the splicing status of many reads and identify the true gene origin of multi-gene mapped reads.

**Availability and implementation:**

Forseti and the code used for producing the results are available at https://github.com/COMBINE-lab/forseti under a BSD 3-clause license.

## 1 Introduction

Single-cell RNA-sequencing (scRNA-seq) technology has revolutionized our understanding of cellular heterogeneity and differentiation dynamics (Stark *et al.* 2019), and short-read, 3′, tagged-end technologies have dominated contemporary data generation. In the most popular scRNA-seq protocols, oligo(dT) primers are used to capture the polyA tail of polyadenylated RNAs. However, recent studies have shown that intronic reads usually account for ∼20% to ∼40% of the total gene count [i.e. distinct number of unique molecular identifiers (UMIs)] in scRNA-seq data (10x Genomics 2021, He *et al.* 2023), suggesting that, in addition to polyA tails, internal adenine-single nucleotide repeats (A-SNR or polyA)—predominantly on unspliced transcripts—are also frequently primed by oligo(dT) primers in scRNA-seq to generate sequenced scRNA-seq reads (Nam *et al.* 2002, 10x Genomics 2021, Svoboda *et al.* 2022). Prior work has also shown that the information captured from unspliced transcripts can offer unprecedented insights into single-cell biology from a brand new perspective (10x Genomics 2022b, Gorin *et al.* 2023, Pool *et al.* 2023, Chamberlin *et al.* 2024, He *et al.* 2024). For example, single-cell RNA velocity (La Manno *et al.* 2018) infers the cellular differentiation dynamics by proposing and performing inference in a model that uses the spliced and unspliced scRNA-seq reads separately to infer the transcriptional dynamics of the underlying genes and cells. This innovation extended the horizon of single-cell data analysis, and has inspired a plethora of subsequent works (Bergen *et al.* 2020, Li *et al.* 2023).

Although the community has been paying increasing attention to the development and use of novel algorithms that utilize information captured from unspliced transcripts, a fundamental problem has yet to be solved. Specifically, the accurate identification of the splicing status of scRNA-seq reads remains a difficult challenge (La Manno *et al.* 2018, Eldjárn Hjörleifsson *et al.* 2022, He *et al.* 2022, 2023). In general, throughout this work, we will refer to “unspliced” molecules in the understanding that they may be actively undergoing splicing and, hence, be partially spliced. It may ultimately be more accurate to refer to these classes of molecules as those with “evidence of unspliced structure” versus those with “no evidence of unspliced structure,” but here we adopt the “spliced”/“unspliced” terminology to remain consistent with the wording used in other mainstream scRNA-seq data processing work.

Currently, the standard strategy of assigning splicing status to reads follows the heuristics introduced in La Manno *et al.* (2018), in which fully and partially intronic reads are classified as unspliced reads, and reads that are only compatible with exonic regions are classified as spliced reads. However, this strategy implies a strong preference for classifying reads as spliced. This is because reads that are entirely contained within the body of an exon, which can originate from either spliced or unspliced transcripts, are all classified as spliced reads. An alternative strategy is to assign an ambiguous splicing status to these exonic reads, indicating that the actual splicing status of the reads is undetermined (Eldjárn Hjörleifsson *et al.* 2022, He *et al.* 2023). However, as a tremendous fraction of scRNA-seq reads are exonic (10x Genomics 2021), applying this strategy leads to, on average, over half of the gene counts (UMIs) being assigned as ambiguous (∼46% to ∼62% for the eight datasets processed in He *et al.* (2023), with a mean of ∼53%).

In this work, we introduce Forseti, the first probabilistic model of which we are aware for resolving the splicing status for *exonic* scRNA-seq reads. Our model does this by taking advantage of the technical details of the underlying scRNA-seq technologies. Specifically, our model is based on the fact that the expected priming sites of oligo(dT) primers should contain an A-SNR or polyA stretch, and the synthesized cDNAs are fragmented with a length preference of ∼300 to ∼400 bp (https://kb.10xgenomics.com/hc/en-us/articles/360000939852-What-is-the-difference-between-Single-Cell-3-and-5-Gene-Expression-libraries-), and follow a distribution that is well-concentrated about the mean. By utilizing the fragment length distribution computed from publicly available, paired-end 3′ scRNA-seq datasets, and a multilayer perceptron (MLP) model trained using the priming sites obtained from these datasets, Forseti obtains a mean Area Under the Receiver Operating Characteristic Curve (AUC) score of 0.92 on simulated data, and 0.88 on the experimental data.

We believe that this work represents a substantial and important step in improving the specificity and accuracy of gene quantification, and specifically splicing status determination, from scRNA-seq data. After describing and evaluating our model to demonstrate its utility, we discuss how the predictions of such a model might immediately aid in improving the downstream processing of scRNA-seq data, but also how the probabilistic allocations produced by our model may, themselves, help to enable more accurate and robust processing, as well as how the model might be extended and enhanced in the future.

## 2 Materials and methods

### 2.1 Data description

In this study, we collected 10 publicly available scRNA-seq datasets using 10× Chromium v2 and v3 solutions for training and testing our model. These datasets consist of both nucleus and cell samples from human and mouse across multiple tissue types (Supplementary Table S1). All these datasets are generated using the alternative sequencing formats, by which the read 1 is sequenced with the same number of cycles as read 2, and therefore, contains the sequence from both ends of the corresponding cDNA insert (10x Genomics 2022a). Compared with the standard sequencing formats, in which read 1 contains only the technical barcode sequences (cellular barcodes and unique molecule identifiers), the reads from the datasets used in this work can be processed as “paired-end” datasets, so as to be used to calculate the cDNA fragment lengths in scRNA-seq by computing the distance between the alignments of paired-end reads (Section 2.3). We processed all human datasets against the GRCh38 version 2020-A genome build and all mouse datasets against the mm10 version 2020-A genome build. Both genome builds were downloaded from the 10x Genomics website (https://support.10xgenomics.com/single-cell-gene-expression/software/downloads/7.0/). We randomly split the ten datasets into eight training and two test datasets (marked in the *train/test* column in Supplementary Table S1). We used only the training datasets to train our model components—the cubic spline and the MLP (Sections 2.4 and 2.5). The two test datasets were used for experimental evaluation (Section 2.7).

### 2.2 Augmented gene annotations

In this work, we built augmented gene annotation sets for mouse and human, which we denote as transcript-level *spliced+unspliced* references, or *spliceu* in short, to obtain the read compatibility to both spliced and unspliced transcripts of genes (Supplementary Section SA.1). The gene annotations were downloaded together with the genome builds (Section 2.1). A *spliceu* reference contains both the spliced and unspliced representations for each transcript annotation. The spliced transcripts of genes were defined as the concatenation of the corresponding exons, and each unspliced transcript was defined as the underlying (genomic) span of all exons of the transcript. Additionally, we found the A-SNR of length six or greater without mismatch on each transcript. The A-SNR information will be used in data simulation (Section 2.8).

### 2.3 Read processing

The composition of a cDNA fragment in the scRNA-seq technologies we consider here has been clearly explained in 10x Genomics (2022a) and Chen *et al.* (2023). Briefly, in scRNA-seq, from the 5′ end to the 3′ end of a first strand cDNA, apart from all PCR and sequencing primers, consists of a cellular barcode (CB) of length 16 bp, a unique molecule identifier (UMI) of length 10 or 12 bp depending on the chemistry, a polyT sequence of length 32 bp corresponding to the oligo(dT) primer, and a cDNA insert of a various length but with a preference of 190–290 bp. When following the alternative sequencing format (10x Genomics 2022a), the corresponding read 1 consists of CB, UMI, a polyT sequence corresponding to the priming window, and the 5′ end of the cDNA insert. The read 2 sequence consists of the 3′ end of the cDNA insert (i.e. the standard “biological” read2). Throughout this work, we define the part in read 1 s representing the CB and UMI sequence as technical read 1 s, and the cDNA insert in read 1 s as biological read 1 s. The detailed procedure of splitting observed, full-length read 1 s into technical read 1 s and biological read 1 s is discussed in Supplementary Section SA.2.

For each dataset, we aligned the sequencing reads twice, each time we wrote both their genome- and *spliceu* transcriptome-based alignments. The detailed procedure of aligning reads can be found in Supplementary Section SA.3. Briefly, we first aligned biological read 1 s and read 2 s as paired-end reads. The resulting alignments were used to train the model components (Sections 2.4 and 2.5). Then, for those reads that can be assigned a definitive splicing status from their pair-end end alignment but not from their read 2 alignment, we aligned only their read 2 to mimic the alignments we would get from a standard scRNA-seq experiment, where only read 2 s represent the corresponding cDNA insert. The resulting alignments were used to evaluate the performance of Forseti (Section 2.7).

### 2.4 cDNA fragment length distribution

We define the cDNA fragment length of a scRNA-seq read as the length of the corresponding cDNA insert. Given a *genome* alignment of a biological read 1, read 2 pair, its corresponding cDNA fragment length is calculated as the contiguous genomic span from the 5′-most genomic locus to the 3′-most genomic locus of the paired-end read alignment. Here, we briefly describe the procedure for fitting the cDNA fragment length distribution. The detailed procedure is discussed in Supplementary Section SA.4. We first calculated the cDNA fragment length of each paired-end read uniquely aligned on the genome. Next, we calculated the frequency of all fragment lengths ranging from 1 to 1000 bp and normalized the frequencies to get the discrete empirical fragment length distribution. We excluded fragment lengths over 1000 bp because the expected fragment length ranges from 190 to 290 bp (Section 2.3), and fragments much longer (i.e. >1000 bp) are expected to be exceedingly rare given the underlying protocol. Finally, we fit the empirical fragment length distribution into a cubic spline. The smoothing condition parameter of the spline was chosen by visual inspection of the training set to balance the fidelity and smoothness.

We note here that we train the fragment length model by identifying fragments where both reads confidently map to the genome and the implied fragment length is ≤1000 bp. This is done *independent* of any annotation. This implies that training data where read 1 and read 2 reside on separate exons, with read 1 primed on the second exon, and where the exons are separated by >1000 bp of intronic sequence, will be ignored or missed in our training. This, of course, may have a negative impact on the prediction accuracy of the model. In fact, incorporating such types of fragments into training and accounting, via an annotation, where such fragments may constitute a proper training example with respect to the annotation is an interesting direction for future work. However, such fragments are likely to be quite rare, and including them will also require that we take care not to “overfit” any specific annotation when incorporating such fragments into the training process. Thus, we leave the consideration of such fragments during training to future work.

### 2.5 The oligo(dT) binding affinity model

A benefit of having biological read 1 s’ alignments is that we can easily extract the downstream sequence of biological read 1 s’ alignments, corresponding to the empirical priming window, from the genome, together with random background sequences (Supplementary Section SA.4). We used these priming windows and background sequences to train a MLP, as discussed in Supplementary Section SA.5. By training an MLP model on the priming windows, it should learn the sequence motifs present therein, and be able to predict the binding affinity of oligo(dT) primers for any given putative priming window. Furthermore, because oligo(dT) primers are designed to capture RNAs without bias, the learned MLP should be generalized to all oligo(dT)-based scRNA-seq data, such as those from 10× Chromium assays.

The MLP is trained and tested using the eight training and two test datasets specified in Section 2.1, respectively. Because of the potential mislabeled examples in the extracted sequences (as discussed in the paragraph below), we first filtered out the extracted priming windows that do not contain an A-SNR of length at least 6, with at most one mismatch. Next, we trained an MLP using the filtered priming window sequences in batches, since the training sets are too large to load into memory at once. During this procedure, we held out 1000 examples from each training dataset as its hold-out set for evaluation. We then tested for overfitting of the trained MLP by comparing the mean prediction accuracy of the MLP on the eight training sets, the hold-out sets from the training datasets, and the two test sets. Our results (Section 3.1) suggest that the trained MLP can predict the hold-out set and the test datasets consistently well, with a mean accuracy of 0.84 and 0.89.

We note that potentially mislabeled sequences in the training data might limit the performance of the trained MLP. There are two types of mislabeling possible in our training data. The first is background sequences that have the potential to be primed but were not selected for priming (these can represent false negatives). The second is intergenic sequences extracted according to the genome alignment of biological read 1 s, but where the priming truly occurs in the polyA tail of the transcribed molecule (these can represent false positives). This can occur because we extracted the priming window corresponding to each read from the genome, while the targets of oligo(dT) primers are RNAs.

### 2.6 The Forseti model

In the evaluation of the splicing status of scRNA-seq reads, a critical piece of potentially useful evidence that is currently unused is the likelihood that particular priming sites give rise to the reads. For example, since the priming of sequenced fragments is expected to originate from the priming to A-SNRs along transcripts, and since fragment lengths in scRNA-seq follow a well-characterized distribution that can be inferred from observed data, one can evaluate the likelihood that a particular A-SNR gives rise to an observed read based on the mapping location of its read 2. Specifically, for a read *r* with a given mapping on a transcript *t*, starting at position *x^t^*, one can consider this read to derive from the end of a cDNA fragment whose opposite end terminates proximate to a downstream A-SNR (i.e. the priming event associated with this fragment). To evaluate the probability of these potential cDNA fragments, one can evaluate the binding affinity of the A-SNR and the probability of observing a cDNA fragment of the implied length under the empirical fragment length distribution. If, for example, it is highly likely that a read is paired with an A-SNR located within an intron, then this provides strong evidence that the associated UMI should be assigned an unspliced status. On the other hand, if the splicing status of this read is fundamentally ambiguous, for example, if the read is likely paired with the poly-A tail and the entire cDNA fragment arises from the region consisting of the terminal exon, 3′ UTR and polyA tail, which are shared by both spliced and unspliced transcripts, the pairing is not particularly informative as to the associated UMI’s splicing status. Fundamentally ambiguous reads are not targeted by Forseti (Section 2.8), and will be (correctly) assigned as ambiguous by the model.

More formally, let the fragment length distribution be *f*. This is a probability distribution that assigns a probability $p_{f}(\ell )$ of observing a fragment of length ℓ. Let the binding affinity distribution *b* be another probability distribution that assigns a binding probability $p_{b}(w)$ for a potential priming window *w* containing an A-SNR. Further, for the start site *x^t^* of a read *r* mapping on transcript *t* of gene *g*, let $\text{pbs}\left(t,x^{t}\right)=\{(w_{1},d_{1}),(w_{2},d_{2}),\ldots \}$ denote the function that returns *all* potential priming windows downstream of *x^t^* in *t* that contains an A-SNR, and, for each such window *w_i_*, the corresponding distance *d_i_* from *x^t^*. Given a read *r* of ambiguous status that maps to position *x^t^* on exon *e* of transcript *t*, we can evaluate the probability that this read arises from *t* (here, defined as the probability of the *most likely* associated binding event) as:

(1) $$\mathrm{Pr}(t|x^{t})=\underset{(w_{i},d_{i})\in \text{pbs}\left(t,x^{t}\right)}{\max}p_{f}(d_{i})\times p_{b}(w_{i}).$$

If, in addition to *t*, the read *r* is compatible with other transcripts of the gene *g*, we consider these as follows. We define a function tx(v,r,g) to be a function that returns *all* transcripts of *g* that are compatible with *r* with a provided splicing status v∈{s,u}, where *s* stands for spliced and *u* stands for unspliced. We can evaluate the probability that a read *r* arises from a transcript of status *v* of gene *g* as:

(2) $$\mathrm{Pr}(v,r,g)=\underset{t_{j}\in \text{tx}(v,r,g)}{\max}\mathrm{Pr}(t_{j}|x^{t_{j}}).$$

Finally, considering both *s* and *u*, the probability that the splicing status of a read *r* is *s* with respect to gene *g* is:

(3) $$\mathrm{Pr}(s|r,g)=\frac{\mathrm{Pr}(s,r,g)}{\sum_{v\in \{s,u\}}\mathrm{Pr}(v,r,g)}.$$

Additionally, if the read *r* maps to multiple genes $G=\{g_{1},g_{2},\ldots \}$, then the most appropriate gene origin *g* for explaining the read is defined as the gene that has the transcript yielding the maximum P(v,r,g) among all genes in *G*, where v∈{s,u}, i.e.

(4) $$g=\underset{g_{k}\in \{G\}}{\text{argmax}}\mathrm{Pr}(v,r,g_{k}).$$

### 2.7 Evaluation on experimental samples

In the experimental data on which we evaluated our model, we extracted sequencing reads for which read 2, when aligned alone, is of ambiguous splicing status, but when paired with biological read 1, the splicing status is determined (or almost certainly determined). In other words, we want to find the read pairs such that the read 2 is entirely contained within an exon but the biological read 1 is not entirely contained within the same exon. Specifically, biological read 1 could be intronic (i), span an intron-exon junction (i·e), span an exon-exon junction (e·e′), or be contained in a *different* exon than read 2 (e${\;}^{\wedge }$e′). In each of these cases, the definitive splicing status, either spliced or unspliced, determined from the biological read 1 served as the ground truth for the subsequent evaluation, where the model, along with the alignments of read 2 treated as single-end data, was used to predict the splicing status of the underlying fragment.

Here, using the alignments for paired-end reads from Section 2.3, we briefly describe the process used to assign a definitive splicing status to reads that have an exonic read 2. We additionally note that reads that have pair-end alignments to multiple genes are ignored in the procedure (i.e. not considered for classification and subsequent evaluation) as we do not know the true gene of origin. First, we used bedtools (Quinlan and Hall 2010) to mark all alignment positions for biological read 1 and read 2 that are entirely contained within exons according to the paired-end alignment records and the exon annotations generated in Section 2.3. Again, if read 2 of a fragment is not contained within any exon, then read 2, by itself, is determinative as to the splicing status and the read pair will not be processed further. We retain the information about whether or not biological read 1 was entirely contained within an exon to aid in subsequent classification.

Given a biological read 1, read 2 pair, we obtain the reference transcripts explaining the paired-end read, denoted as $\left\{T^{\text{pe}}\right\}$, from its alignments. We also obtain the transcripts that are compatible with an *exonic* alignment of biological read 1 and read 2, denoted as $\left\{T_{e}^{r1}\right\}$ and $\left\{T_{e}^{r2}\right\}$, respectively.

We identify the inferred status of the read, as spliced or unspliced, as follows:

- e${\;}^{\wedge }$e′ reads: When (i) $\hat{T}=\{T^{\text{pe}}\}\cap \{T_{e}^{r1}\}\cap \{T_{e}^{r2}\}\neq \emptyset$, (ii) $\hat{T}$ contains only spliced transcripts, (iii) the corresponding exons in each $\hat{t}\in \hat{T}$ that are compatible with biological read 1 and read 2 are different, and (iv) the genomic distance between the exons containing read 2 and biological read 1 in each $\hat{t}\in \hat{T}$ are >1000 nucleotides apart, then we assign the read as a spliced (e${\;}^{\wedge }$e′) read, because this means the fragment (but not either of the individual reads) spans an exon-exon junction of the common reference transcripts. Here, we require the distance between the involved exons to be larger than 1000 nucleotides to minimize mislabelling caused by short introns, where the fragment may place the reads on different exons and completely contain a short intervening intron.
- e·e′ reads: When (i) $\{T^{\text{pe}}\}\cap \{T_{e}^{r1}\}\cap \{T_{e}^{r2}\}=\emptyset$, (ii) $\{T^{\text{pe}}\}\cap \{T_{e}^{r2}\}\neq \emptyset$, and (iii) the intersection contains only spliced transcripts, we assign the read as a e·e′ read, and therefore of spliced status, because this means its biological read 1 spans an exon-exon junction.
- i and i·e reads: When (i) $\{T^{\text{pe}}\}\cap \{T_{e}^{r1}\}\neq \emptyset$ and (ii) the intersection contains only unspliced transcripts, we assign the read as an unspliced read because this means its biological read 1 either crosses an intron-exon junction (i·e) or is complete contained within an intron (i), and therefore the read pair must have arisen from an unspliced molecule.

We processed each read with an exonic read 2 individually to get the spliced and unspliced labels for the evaluation set. We then applied our model using the alignments of read 2 s, treated as single-end data, to predict the gene origin and the splicing status of the read. The model prediction of reads was compared with their assigned labels according to the above criteria to evaluate the performance of the model.

### 2.8 Data simulation

In addition to the experimental data, which employ a collection of rules involving the paired-end mapping of read 1 and read 2 to ascertain the true splicing status, we also evaluated our model on simulated data, where the true splicing status of each fragment is known with certainty. As with all simulations, the trade-off here is that the simulated data is, in general, simpler and “cleaner” than the experimental data. We emphasize that splice-aware single-cell read simulation is an ongoing research task, and our simulation, although not perfect, is, to our knowledge, the first algorithm that can simulate scRNA-seq reads according to provided spliced and unspliced read counts.

We simulated paired-end reads using the human *spliceu* reference (Section 2.2). In order to mimic real data at both read-count and read-sequence levels, we seed our simulation from experimental data and introduced realistic sequencing errors into the simulated reads. Specifically, we processed a 10X 1k Human PBMC v3 scRNA sample (https://www.10xgenomics.com/datasets/1-k-pbm-cs-from-a-healthy-donor-v-3-chemistry-3-standard-3-0-0) using simpleaf (He and Patro 2023) with the *spliceu* reference to obtain the spliced, unspliced, and ambiguous counts of each gene in each cell (Supplementary Section SA.6).

As detailed in Section 2.2, regions on the reference sequence with at least six consecutive adenine (A) bases were considered potential polyA priming sites. We denote by $\vec{t_{A}}$ the ordered list of potential polyA priming sites on transcript *t*. The polyA tail of each transcript was also considered a potential priming site.

Simulated reads were generated from the spliced and unspliced transcripts recorded in *spliceu* according to the following process. First, a gene *g* is selected for sequencing. For each gene *g* we draw *g*_s_ spliced reads and *g*_u_ unspliced reads, where *g*_s_ and *g*_u_ are the corresponding counts from the 10X PBMC dataset. Given a gene *g* and a read status v∈{u,s}, we randomly selected a transcript *t* of *g* with the desired splicing status *v*. Next, we selected a polyA site from $\vec{t_{A}}$ uniformly at random; let the site be denoted as $t_{A_{i}}$. Then, we sampled a fragment length *d* according to the empirically derived fragment length distribution *f*. The pair of $\left(t_{A_{i}},d\right)$ determines the underlying fragment being sampled, which spans the transcriptomic region of length *d* ending at $t_{A_{i}}$. This determines the position of read 2 (the first 91 bases at the 5′) as well as the corresponding read 1 (the last 31 bases at the 3′), matching the cycle length of the reference dataset.

As our focus in this work is on recovering the splicing status of fragments, the only simulated reads useful for our experiments are those where read 2 resides entirely within an exon. So as to not waste effort simulating and quantifying fragments where read 2 is, itself, of determined splicing status, we exclude fragments having a non-exonic read 2 from our simulated data. Thus, during simulation, we inspect each simulated potential fragment to determine if read 2 is ambiguous or not. If read 2 itself is definitive of the splicing status of the underlying molecule, we discard this read and sample a fragment from the same gene and splicing status. To achieve this while still striving to obtain the desired read count for each gene, we implemented a resampling process, limited to a maximum of 100 000 attempts per read.

We note that, because we rejected and resampled “naturally simulated” fragments for which read 2 had a determinative splicing status, there is a selection effect among the fragments that eventually complete the simulation process, and that the simulated data is therefore not entirely random with respect to the underlying collection of polyA sites and the target fragment length distribution. Nonetheless, we found that this process performs reasonably well in terms of generating fragments whose ambiguity profiles match those of experimentally ambiguous fragments.

Moreover, it is also important to note that two specific categories of reads are *fundamentally ambiguous* and remain indistinguishable within our model and, in fact, in the context of any model that just considers the type of information used in Forseti. The first category includes fragments where read 1 is from the polyA tail and read 2 resides in the terminal exon of the transcript of origin. Such fragments can originate from either the spliced or unspliced versions of a transcript, leading to inherent and fundamental ambiguity. The second category comprises read pairs where the whole fragment (read 2 and its mate read 1) are from *the same exon* within a transcript. These are *fundamentally ambiguous* for the same reason as the first category but tend to be rare in practice as typical fragment lengths are longer than typical exon lengths. To test the performance of our model with and without fundamental ambiguity, on top of the data simulated by the procedure described above, we also simulated a read set without fundamental ambiguity. In this set, we require not only that read 2 of a fragment is contained in an exon, but also that its corresponding read 1 is not contained within the same exon, and the fragment is not generated from the polyA tail of transcripts.

To mimic realistic sequencing error profiles, we employed InSilicoSeq (ISS) (Gourlé *et al.* 2019) to introduce realistic Illumina errors into the simulated reads (Supplementary Section SA.6).

## 3 Results

**Figure 1.**
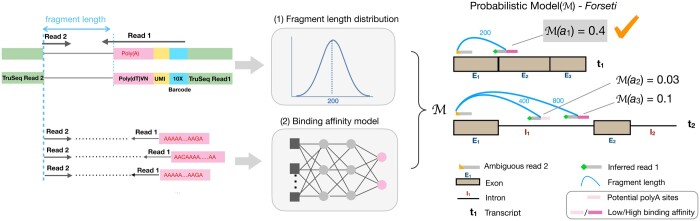
Overview of the Forseti model of splicing status inference. The fragment length and binding affinity models are used to score putative fragments, and the splicing status of a molecule giving rise to the highest-scoring fragment yields the prediction.

In this study, we developed a probabilistic model, Forseti, to infer the splicing status of the molecule origin for scRNA-seq reads (Fig. 1). Our model makes use of a fragment length distribution and a binding affinity model learned from empirical data to identify and score the potentially sequenced fragments that led to the observed scRNA-seq reads. From these scored fragments, Forseti can not only predict the splicing status of the molecule origin that the read was drawn, but also recognize the true gene origin when reads are compatible with multiple genes. We evaluated Forseti on both simulated and experimental validation sets, and found that Forseti precisely predicts the splicing status of reads in all validation datasets, with AUC scores ranging from 0.85 to 0.93 and an average AUC of 0.90.

### 3.1 Evaluation of model components

**Figure 2.**
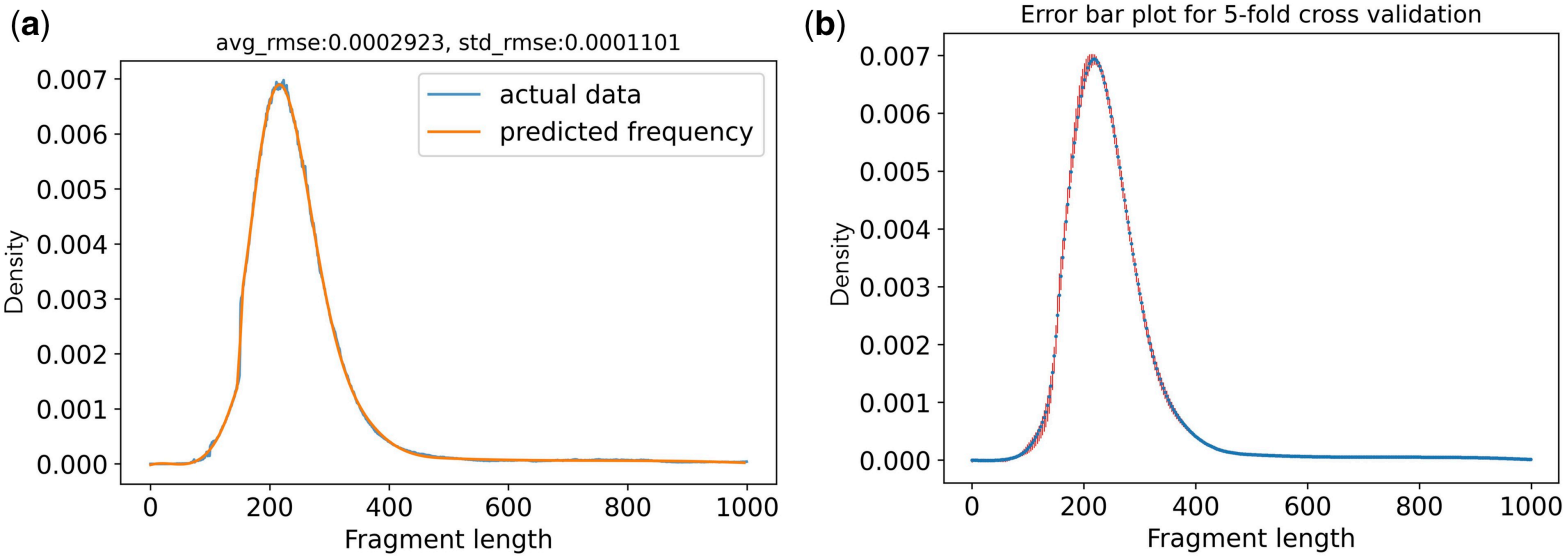
The fragment length spline model fits the actual data well. Panel (a) shows the accuracy of the spline model on test datasets, measured by the Root Mean Square Error (RMSE) score. The blue line represents the empirical fragment length distribution of the two test datasets. The orange line represents the corresponding spline model predictions. Panel (b) shows the variance (error bars) of the trained spline models from each of the 5-fold cross-validation.

In most tagged-end scRNA-seq protocols, the cDNA fragments are generated with a preferred length of 300–400 bp, which means the cDNA fragment length in scRNA-seq might follow a distribution. However, the potential cDNA distribution is challenging to model, or even detect, in most existing scRNA-seq data, because the recommended sequencing format captures only the 3′ end sequence of the cDNA insert. To overcome this challenge and model the cDNA fragment length distribution, we collected 10 scRNA-seq datasets that were generated by applying the alternative sequencing format (10x Genomics 2018), in which read 1 is sequenced the same cycles as read 2, so that the sequence of the 5′  *and* 3′ end of the cDNA insert in each sequenced fragment are both captured, by read 1 and read 2, respectively. In other words, these data can be treated as paired-end data, and the cDNA fragment length of each sequenced fragment can be calculated from the alignments of the paired-end reads. In this work, we fit a cubic spline model on the empirical fragment lengths calculated according to the paired-end read alignments from the eight training datasets selected from the collected datasets (Supplementary Table S1) and evaluated its performance on the two holdout testing datasets (Section 2.4). As shown in Fig. 2a, the fragment length distribution model’s close fit to data strikes a balance between smoothness and fidelity. The low Root Mean Square Error (RMSE) score in our evaluation indicates a high accuracy of the model in predicting the fragment length probability within the two test datasets (mean RMSE mean is lower than $3\;\times \;e^{-4}$ and RMSE standard deviation is lower than $1\;\times \;e^{-4}$). In addition, we assessed the spline model’s generalizability with a 5-fold cross-validation experiment. For each fold, we trained and evaluated a cubic spline model with a random training, test split. The error bar plot in Fig. 2b illustrates that minimal variation is observed in the fragment length probability predictions across all 5-folds, indicating model’s consistent performance and a generally similar fragment length distribution across samples. This consistency also supports our initial hypothesis regarding a generalized insert size distribution and that a distribution can be learned and robustly applied to various datasets across different species, cycle lengths, and chemistry versions.

In addition to the fragment length model, Forseti utilizes a MLP to predict the binding affinity of potential priming windows containing the sequence motif where the oligo(dT) primers bind. The MLP was trained and tested on experimental priming windows obtained from the eight training and two test datasets (Supplementary Table S1). The mean accuracy of the trained MLP on the training datasets, the holdout sets from the training data, and the tested datasets is 0.83, 0.84, and 0.89, respectively, suggesting the high accuracy and generalizability of the trained MLP for identifying the experimental priming windows from random background sequences. Per-dataset mean accuracy scores are listed in Supplementary Table S2.

### 3.2 Forseti accurately predicts the splicing status of reads

We evaluated Forseti on four evaluation read sets, two from experimental data, and the other two from simulated data (Sections 2.7 and 2.8). Because Forseti, and in fact, any model that just uses the same information, are unable to predict a definitive splicing status for *fundamentally ambiguous* fragments—fragments arising either from polyA tail priming and staying in the terminal exon, or having both biological read 1 and read 2 entirely contained within the same exon—we generated two sets of simulated data; one with and one without fundamental ambiguity (Section 2.8). In this section, we ignored the reads predicted as ambiguous by Forseti when evaluating the performance. The results including ambiguous predictions (dominated, in fact, by *fundamentally ambiguous* fragments), are provided and discussed in Supplementary Section SB.

**Figure 3.**
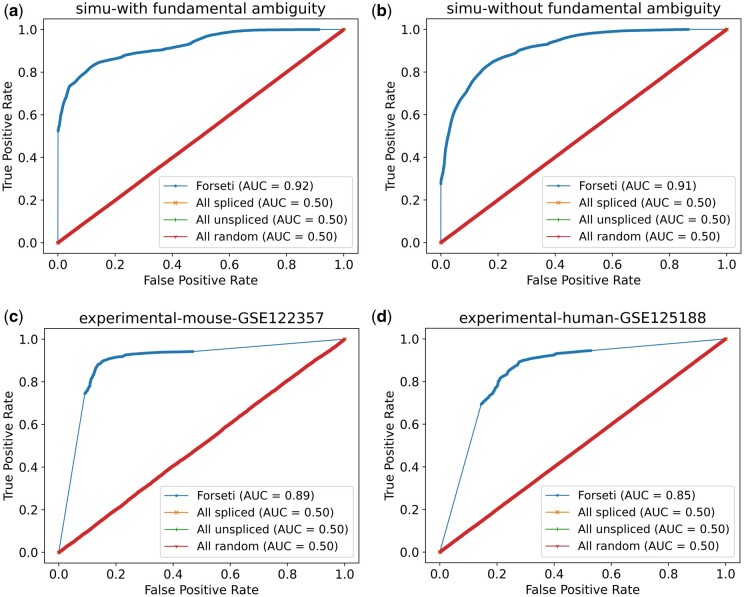
Forseti accurately predicts the splicing status of both simulated and experimental reads, evaluated using receiver operating characteristic (ROC) curve and Area Under ROC (AUC) score. In all plots, the blue curve represents the ROC of Forseti, and the other three curves stacking with each other, represent the three baseline models. Panel (a) and (b) show the ROC curve and the corresponding Area Under ROC (AUC) of simulated with and without fragments entirely contained within an exon and fragments arising from terminal polyA priming, respectively. Panel (c) and (d) show the AUC of the two experimental datasets from mouse and human, respectively.

The model performance was assessed using the receiver operating characteristic (ROC) curve and, specifically, by evaluating the area under ROC curve (AUC) scores. The ROC curve plots the True Positive Rate (TPR) and False Positive Rate (FPR) from all predictions as the classification threshold is swept across the value of all scores. The number of spliced and unspliced reads and the TPR and FPR of Forseti predictions on those sets are provided in the Supplementary Table S3. The AUC, ranging from 0 to 1, measures the area under the ROC curve. A high AUC score means a model has a good prediction power—that the probability assigned by our model to a splicing status is well-correlated with the true splicing status. As shown in Fig. 3, our model demonstrated consistently high AUC among all evaluated read sets. In particular, the AUC of Forseti on the two experimental sets generated from mouse and human scRNA-seq datasets is 0.89 and 0.85 (Fig. 3c and d), suggesting the generality of our model across different species and cell types. The high AUC scores of Forseti on the simulated data with and without fundamental ambiguity (Fig. 3a and b), consistent with the experimental sets, suggest that the mechanism—internal polyA priming—we used to generate the simulated dataset (Section 2.8) well-mimics the real-world mechanism that generates ambiguous reads. Also, our result highlights that including fundamental ambiguity did not hamper the ability of our model to predict the correct splicing status for reads when possible. We also included the ROC curve of three baseline models, implying the current best practices for resolving splicing ambiguity in Fig. 3 (La Manno *et al.* 2018, Kaminow *et al.* 2021, 10x Genomics 2022b; Eldjárn Hjörleifsson *et al.* 2022, He *et al.* 2022). These baseline methods either assign all reads a spliced status probability of 1 (“All spliced”), 0 (“All unspliced”), or a random probability ranging from 0 to 1 (“All random”). This “All random” predictor is generated by first assigning a random probability in [0,0.5] to each truly unspliced read and a random probability selected in [0.5,1] to each spliced read, and then randomly shuffling the order of these probabilities. All three baseline models had an AUC = 0.5, suggesting that no trivial predictor can extract meaningful splicing status at a level approaching that achieved by Forseti.

### 3.3 Forseti can disambiguate gene multimapping reads

One advantage of being a probabilistic model is that the predicted probabilities of Forseti can be regarded as the confidence score that each potential downstream priming site, when paired with an alignment for read 2, represents that actual underlying fragment from which read 2 was sequenced. The final splicing status of a read with respect to a gene is assigned as the splicing status of the reference transcript for which the most confident alignment, priming site pair occurs. Similarly, this highest confidence score (probability) can *also* be used to evaluate the true gene origin of reads compatible with multiple genes (which may arise because of overlapping genes in the genome, or because of sequence-similar genes, such as members of a gene family).

To select a splicing status among spliced and unspliced transcripts within a gene, Forseti simply compares the highest-scoring fragment from any spliced transcript to the highest-scoring fragment from any unspliced transcript. However, the same underlying predictive scoring mechanism can also be used to evaluate the relative probability of the truly generating fragment *between* genes.

Specifically, consider a read that is multimapping between two genes *g*_1_ and *g*_2_. For each gene, Forseti computes a score for all potential fragments and records the highest score among all transcripts of *g*_1_ and all transcripts of *g*_2_, respectively. Then, we can compare the highest score computed for *g*_1_ and *g*_2_ to determine the best gene origin of the read between these genes. In the current work, we consider the maximum score as the criterion when comparing compatible genes. However, we note that other aggregation mechanisms, such as the sum, the expected fragment probability, or a weighted expectation, can also be used for scoring genes, and evaluating optimal aggregation mechanisms is an interesting direction for future work.

One finding from our result is that, in our simulated data, multi-gene mapped reads were all simulated from unspliced transcripts, mainly from the regions that are shared by multiple genes. Because the sequence of these shared regions is identical, the true gene origin of these reads is indistinguishable in our model. However, for the two experimental evaluation datasets, Forseti assigned a single best gene origin to 65% of the multi-gene mapped reads in one dataset and to 93% of the multi-gene mapped reads in the other (the remaining reads had a tied highest score among genes, and thus no single gene prediction could be made). However, when a single gene prediction could be made, Forseti’s prediction was correct in 75% and 69%of the cases, respectively (i.e. the true gene origin of the multi-gene mapped reads was identified). This suggests that in reality, most multi-gene mapped reads are distinguishable, and, among the distinguishable subset of multi-gene mapped reads, Forseti can assign the true gene origin to them in the majority of cases. As the gene origin assignment is affected by the weight Forseti places on internal polyA compared to polyA tail priming, a weight parameter naturally arises that affects the relative preference for polyA tails versus internal polyA priming. Exploring the trade-off between the accuracy of gene-origin prediction and splicing status prediction by optimizing or altering this weight is an interesting direction for future work. Here, however, we consider just the simple case where we treat the two polyA classes equally.

While the rescue of multi-gene reads is not the primary focus of our model, and was not a target application during Forseti’s design, the fact that it provides meaningful predictions in this distinct task is evidence of the promise that fundamentally better fragment modeling can make to scRNA-seq read processing. To our knowledge, our model is the first to attempt to resolve fragment-level multi-gene mapping in scRNA-seq data, thanks to the utilization of the fragment length distribution and binding affinity models. Further, these informative predictions stack on top of existing models for partially allocating gene-multi-mapping UMIs, such as the EM approach introduced in Srivastava *et al.* (2019) and later adopted in Melsted *et al.* (2021), Kaminow *et al.* (2021), and He *et al.* (2022).

## 4 Conclusion

In this work, we introduce Forseti, a mechanistic and predictive model of the splicing status of the sequenced fragments in scRNA-seq samples. As the first predictive model of which we are aware that focuses on resolving the splicing status ambiguity in scRNA-seq, Forseti utilizes the fragment length distribution and binding affinity models trained on a variety of experimental scRNA-seq datasets to predict the probability that the observed read is associated with specific sequenced fragments arising from each compatible reference transcript. Our results show that the AUC of our model on both experimental and simulated evaluation sets is high, demonstrating the consistent performance of Forseti across species and cell types. Furthermore, by virtue of being a probabilistic model that seeks to score potential fragments of origin of a read, and not *just* the read’s splicing status, our model predictions can also be compared across genes and used to help successfully resolve the gene of origin for the majority of multi-gene mapped reads in our experimental evaluation datasets.

Because Forseti predicts the splicing status of sequenced fragments based on the difference of the nucleotide sequence of the spliced and unspliced transcripts, one limitation of Forseti is that, when the possible fragments in spliced and unspliced transcripts are identical (for example for fragments are entirely contained within an exon) Forseti will be unable to resolve the splicing status of the fragments. However, one potential path to overcoming this limitation is to aggregate evidence over all fragments from the same unique molecule identifier. In this case, an individual read maybe *fundamentally ambiguous*, but other fragments of the same UMI may have a definitive splicing status or at least an informative splicing probability, so as to be used to distinguish the splicing status of their common molecule of origin. Likewise, another potential use case for Forseti at the UMI level is to aid in determining potential UMI collisions. If a UMI contains conflicting evidence, i.e. some of its sequenced fragments are predicted as spliced and others as unspliced, this might indicate UMI collision, suggesting the potential of using Forseti to also improve the UMI resolution in scRNA-seq. Finally, we have developed Forseti as a proof-of-concept model, demonstrating the potential and promise of fragment-level modeling to elucidate splicing status in scRNA-seq data. However, one important direction for future work is to properly integrate this model into existing tools for efficient scRNA-seq processing, like our alevin-fry tool (He *et al.* 2022). While not conceptually difficult, such integration will require the propagation of additional information through the processing pipeline, and it will also likely highlight the need and opportunity for further computational enhancements and simplifications that will make the fragment probabilities evaluated in Forseti faster to calculate at the scale of ever-growing scRNA-seq experiments.

## Supplementary Material

btae207_Supplementary_Data

## Data Availability

All the datasets are publicly available and can be downloaded from NCBI GEO at https://www.ncbi.nlm.nih.gov/geo/. The GSE numbers are listed in the Supplementary Table S1. In addition, the data used in the simulation step are available in 10X Genomics at https://www.10xgenomics.com/datasets/1-k-pbm-cs-from-a-healthy-donor-v-3-chemistry-3-standard-3-0-0.

## References

[btae207-B1] 10x Genomics. 2018. Technical Note—Base Composition of Sequencing Reads of Chromium Single Cell 3′ v2 Libraries, Document Number CG000080, 10x Genomics (November 19, 2018). https://cdn.10xgenomics.com/image/upload/v1660261285/support-documents/CG000080_10x_Technical_Note_Base_Composition_SC3_v2_RevB.pdf

[btae207-B2] 10x Genomics. 2021. Technical Note—Interpreting Intronic and Antisense Reads in 10x Genomics Single Cell Gene Expression Data, Document Number CG000376, 10x Genomics (August 9, 2021). https://cdn.10xgenomics.com/image/upload/v1660261286/support-documents/CG000376_TechNote_Antisense_Intronic_Reads_SingleCellGeneExpression_RevA.pdf

[btae207-B3] 10x Genomics. 2022a. Technical Note—Assay Scheme and Configuration of Chromium Single Cell 3’ v2 Libraries, Document Number CG000108, 10x Genomics (December 2, 2022). https://cdn.10xgenomics.com/image/upload/v1660261286/support-documents/CG000108_AssayConfiguration_SC3v2.pdf

[btae207-B4] 10x Genomics 2022b. Technical Note—Interpreting Single Cell Gene Expression Data With and Without Intronic Reads, Document Number CG000554, 10x Genomics (June 21, 2022). https://cdn.10xgenomics.com/image/upload/v1660261285/support-documents/CG000554_Interpreting_SingleCellGEX_with_introns_RevA.pdf

[btae207-B5] Bergen V , Lange M, Peidli S et al Generalizing RNA velocity to transient cell states through dynamical modeling. Nat Biotechnol 2020;38:1408–14. 32747759 10.1038/s41587-020-0591-3

[btae207-B6] Chamberlin JT , Lee Y, Marth GT et al Differences in molecular sampling and data processing explain variation among single-cell and single-nucleus RNA-seq experiments. Genome Res 2024;34:179–88. 38355308 10.1101/gr.278253.123 PMC10984380

[btae207-B7] Chen X , Roelli P, Hereñú D et al 2023. Teichlab/scg_lib_structs: Release October 26, 2023. https://zenodo.org/doi/10.5281/zenodo.10042390

[btae207-B8] Eldjárn Hjörleifsson K , Sullivan DK, Holley G et al 2022. Accurate quantification of single-nucleus and single-cell RNA-seq transcripts. 10.1093/nar/gkae1137 PMC11724275 39657125

[btae207-B9] Gorin G , Yoshida S, Pachter L. Assessing Markovian and delay models for single-nucleus RNA sequencing. Bull Math Biol 2023;85:114. 37828255 10.1007/s11538-023-01213-9

[btae207-B10] Gourlé H , Karlsson-Lindsjö O, Hayer J et al Simulating illumina metagenomic data with insilicoseq. Bioinformatics 2019;35:521–2. 30016412 10.1093/bioinformatics/bty630 PMC6361232

[btae207-B11] He D , Patro R. simpleaf: a simple, flexible, and scalable framework for single-cell data processing using alevin-fry. Bioinformatics 2023;39:btad614. 10.1093/bioinformatics/btad614 PMC10580267 37802884

[btae207-B12] He D , Mount SM, Patro R. 2024. scCensus: Off-target scRNA-seq reads reveal meaningful biology. bioRxiv. 10.1101/2024.01.29.577807

[btae207-B13] He D , Soneson C, Patro R. 2023. Understanding and evaluating ambiguity in single-cell and single-nucleus RNA-sequencing. bioRxiv. 10.1101/2023;2023.01.04.522742.

[btae207-B14] He D , Zakeri M, Sarkar H et al Alevin-fry unlocks rapid, accurate and memory-frugal quantification of single-cell RNA-seq data. Nat Methods 2022;19:316–22. 35277707 10.1038/s41592-022-01408-3 PMC8933848

[btae207-B15] Kaminow B , Yunusov D, Dobin A. 2021. STARsolo: accurate, fast and versatile mapping/quantification of single-cell and single-nucleus RNA-seq data. bioRxiv. 10.1101/2021.05.05.442755.

[btae207-B16] La Manno G , Soldatov R, Zeisel A et al RNA velocity of single cells. Nature 2018;560:494–8. 30089906 10.1038/s41586-018-0414-6 PMC6130801

[btae207-B17] Li S , Zhang P, Chen W et al A relay velocity model infers cell-dependent RNA velocity. Nat Biotechnol 2023;42:99–108. 37012448 10.1038/s41587-023-01728-5 PMC10545816

[btae207-B18] Melsted P , Booeshaghi AS, Liu L et al Modular, efficient and constant-memory single-cell RNA-seq preprocessing. Nat Biotechnol 2021;39:813–8. 33795888 10.1038/s41587-021-00870-2

[btae207-B19] Nam DK , Lee S, Zhou G et al Oligo(dT) primer generates a high frequency of truncated cDNAs through internal poly(A) priming during reverse transcription. Proc Natl Acad Sci USA 2002;99:6152–6. 11972056 10.1073/pnas.092140899 PMC122918

[btae207-B20] Pool A-H , Poldsam H, Chen S et al Recovery of missing single-cell RNA-sequencing data with optimized transcriptomic references. Nat Methods 2023;20:1506–15. 37697162 10.1038/s41592-023-02003-w

[btae207-B21] Quinlan AR , Hall IM. BEDTools: a flexible suite of utilities for comparing genomic features. Bioinformatics 2010;26:841–2. 20110278 10.1093/bioinformatics/btq033 PMC2832824

[btae207-B22] Srivastava A , Malik L, Smith T et al Alevin efficiently estimates accurate gene abundances from dscRNA-seq data. Genome Biol 2019;20:65. 30917859 10.1186/s13059-019-1670-y PMC6437997

[btae207-B23] Stark R , Grzelak M, Hadfield J. RNA sequencing: the teenage years. Nat Rev Genet 2019;20:631–56. 31341269 10.1038/s41576-019-0150-2

[btae207-B24] Svoboda M , Frost HR, Bosco G. Internal oligo(dT) priming introduces systematic bias in bulk and single-cell RNA sequencing count data. NAR Genom Bioinform 2022;4:lqac035. 35651651 10.1093/nargab/lqac035 PMC9142200

