# Supplementary material for: Forseti: a mechanistic and predictive model of the splicing status of scRNA-seq reads
Source: Bioinformatics. 2024 Jun 28;40(Suppl 1):i297–306. doi: 10.1093/bioinformatics/btae207 (PMC11256924; doi:10.1093/bioinformatics/btae207)
Supplement: btae207_Supplementary_Data [file btae207_supplementary_data.zip › btae207_Supplementary_Data/Patro.258.supp.pdf]

Appendices

Table 1. Details of the ten datasets used in this work. The *Chemistry* column records the version of the 10x Chromium 3′ solution used for the corresponding datasets. The *train/test* column represents the label of the random split of the datasets used for training and testing `Forseti`. All datasets are publicly available and can be downloaded from NCBI GEO at https://www.ncbi.nlm.nih.gov/geo/.

#	GSE ID	Species	Cell type	Chemistry	Read length	Cells or Nuclei	train/test
1	GSE144136	Human	Brain	v2	100	Nuclei	train
2	GSE148504	Human	Cardiomyocytes	v3	150	Cells	train
3	GSE122743	Human	MCF7	v2	75	Cells	train
4	GSE125970	Human	Ileum/Colon/Rectum	v2	150	Cells	train
5	GSE130636	Human	Retina	v3	150	Cells	train
6	GSE131736	Human	Retina	v2	75	Cells	train
7	GSE134520	Human	Stomach	v2	150	Cells	train
8	GSE135922	Human	RPE/Choroid	v3	151	Cells	train
9	GSE122357	Mouse	Brain	v2	151	Cells	test
10	GSE125188	Human	Liver/Blood/Spleen	v2	150	Cells	test

Appendix A Supplementary Methods

A.1 Details of building the augmented references

A *spliced+unspliced* reference, or *spliceu* in short, contains both the spliced and unspliced transcripts of genes (Section 2.2). The spliced version of each transcript is the concatenation of the corresponding exons. The unspliced version is a contiguous genomic interval from the 5′-most exonic locus to the 3′-most exonic locus of the exons. Single-exon transcripts (mainly non-coding RNAs) do not have their unspliced transcript in *spliceu*, because they do not have introns to be spliced out. We first loaded the 10X genome build (Section 2.1) using the `readDNAStringSet` function from `Biostrings` and the exon by transcript information with `getFeatureRanges` from `eisaR` by passing `spliced` to the `featureType` parameter. The exon annotations were then written as a `BED` file using `export.bed` function from `rtracklayer`.

With the transcript sequences in hand, we also found the A-SNRs on each transcript using the `vmatchPattern` function from `Biostrings`. Here a A-SNR is defined as an adenine-single nucleotide repeat of length 6 or greater without mismatch. The A-SNR information will be used to assist in simulation.

A.2 Details of scRNA-seq read quality control

In order to align biological read 1s and read 2s as paired-end reads, we first segmented each observed read 1 into a technical read 1, which contains the CB and UMI sequences, and a biological read 1 segments, which contains the 3′ tail representing the cDNA insert, in a `Python` (version 3.10.13) environment using `dnaio` (Martin and Vorderman, 2023). Subsequently, to ensure data quality, we used `fastp` (Chen et al., 2018) for filtering out low-quality biological read 1 and read 2 pairs. We set a stringent quality threshold (`qualified_quality_phred` = 30) and a limit on the percentage of unqualified bases (`unqualified_percent_limit` = 30). Additionally, we filtered out low complexity reads, which are usually caused by polyA tail priming and contain a polyA stretch, by setting a low complexity filter (`low_complexity_filter` = 30). Furthermore, a minimum read length of 10 bases was enforced using the `length_required` parameter, and the adapter trimming was disabled.

A.3 Details of aligning scRNA-seq reads

The preprocessed biological read 1 and read 2 pairs (Sections A.2 and 2.3) were aligned using `STAR` (Dobin et al., 2013) (version 2.7.11a). *spliceu* STAR indices were built for mouse and human using their 10X standard genome build and the corresponding *spliceu* reference, respectively. Beyond the default configuration, the parameters passed to STAR are detailed below. Genome-based alignments were exported as a BAM file and sorted by genome coordinate by passing `BAM SortedByCoordinate` to the `outSAMtype` argument. Additionally, a transcriptome-based BAM file was generated by passing `TranscriptomeSAM` to the `quantMode` parameter. We used the `Singleend` option of the `quantTranscriptomeBan` option to allow soft clips and gaps in transcriptome-based alignments. Additionally, we specified `outFilterScoreMinOverLread` and `outFilterMatchNminOverLread` as 0.33 to adjust this length-sensitive threshold, and to output the alignment for the split read 1, which has a shorter sequence than read 2. We set `alignSplicedMateMapLminOverLmate` as zero to allow more alignments in paired-end mapping. For accurate fragment length calculation, we filtered the genome-based alignments of the paired-end reads using `samtools` (Li et al., 2009) to ensure that the reads are properly and uniquely mapped to the genome by specifying `require-flags 2` and `d NH:1` for paired-end alignments. Those filtered reads were then used to run STAR again using single-end mode by passing their read 2s to get the alignments of read 2s only. The resulting alignments were used to evaluate the performance of `Forseti` by comparing the consistency of the splicing status predicted by `Forseti` using read 2s' alignments (predictions) with those from paired-end alignments (ground truths).

A.4 Details of cDNA fragment length distribution

The cDNA fragment length indicated by a scRNA-seq read alignment is defined as the genomic interval spanned by the paired-end read alignment (biological read 1 and read 2). We processed the paired-end alignments (Sections A.3 and 2.3) of the selected datasets in a `Python` version 3.10.13 environment using `biopython` (Cock et al., 2009) and `pysam` [§]. We read the genome FASTA file using the `SeqIO` module from `biopython`. Next, given the genome alignment of a uniquely mapped paired-end read as an `AlignedSegment` (from `pysam`), we found the interval spanned by the paired-end alignment on the genome according to the corresponding `SAM` flags indicating their orientation and the `CIGAR` string. Specifically, we found the distance D between the closer ends of the paired-end alignment. Then, we summed the read length of biological read 1 and read 2, and D to get the final fragment length L. If the biological read 1 and read 2 intersect and they share gaps that are designated as *intronic* by `STAR` (as indicated by the character "N" in the `CIGAR` string), the gaps will not be included when calculating D. Additionally, the genomic sequence of the priming window captured by the oligo(dT) primer of this read, defined as the downstream 30-base genomic interval of biological read 1, was extracted from the genome according to the alignment. We also extracted a background genomic interval randomly selected around biological read 1. The extracted priming windows and background windows are used in Section 2.5 for training the binding affinity model. We emphasize that it is possible that, in some cases, read 1 overlaps the junction between the terminal exon and the polyA tail of a polyadenylated transcript. In such cases, the intergenic sequence downstream of the terminal exon in the genome will be regarded as the priming window, because the polyA tail of transcripts is not included in the genome build. Although we found that most of the priming window sequences contain an A-SNR, we filtered the extracted priming windows according to the existence of A-SNRs to address this potential problem (Section A.5).

Note that the procedure is designed for processing reads generated from internal polyA priming that does not contain a gap (exon-exon junction) in the interval between the paired-end alignment. If the interval between a paired-end alignment, that is not covered by the alignment itself, spans an exon-exon junction, then the final fragment length will contain the length of the gap. If the fragment originates from polyA tail priming and its biological read 1 is a long A-SNR, then most likely, it will not align to the genome, and so the corresponding fragment will not be used in fragment length calculation (as intended), since it does not map as a proper pair. However, our results suggested that these exceptional cases are rare in the selected datasets, as most fragment lengths fall into the expected range, from 190 to 290 base pairs.

We fit the empirical scRNA-seq cDNA fragment lengths obtained above into a cubic spline using `scipy` (Virtanen et al., 2020). As the expected fragment length ranges between 190 to 290 base pairs (Section 2.3), we used all fragment lengths no larger than 1,000 base pairs to fit the spline. Specifically, For each of the eight training datasets (Section 2.1), we calculated the frequency of all fragment lengths ranging from 1 to 1,000 and normalized the frequencies to get the discrete empirical fragment length distribution. Then, we fit a cubic spline on the distribution using `splrep` from `scipy`. The smoothing condition parameter `s` was chosen by visual inspection. We set the smoothing parameter to $1/10^6$ to balance the fidelity and smoothness. Specifically, we train the fragment length model by identifying fragments where both reads confidently map to the genome and the implied fragment length is $< 1,000$ bp. This is done independently of any annotation. We note that this implies that spliced reads, where read 1 and read 2 reside on separate exons, with read 2 primed on the second exon, and where the exons are separated by $> 1,000$ bp of intronic sequence, will be ignored, and then missed, in our training — and this may have a negative impact on the prediction accuracy of the model. In fact, incorporating such types of fragments into training is an interesting direction for future work.

A.5 Details of the oligo(dT) binding affinity model

In section A.4, we extracted the downstream sequence of biological read 1 corresponding to the empirical priming window together with background sequences. We then used these sequences to train a multi-layer perceptron (MLP) using `MLPClassifier` from `sklearn` (Pedregosa et al., 2011). By training an MLP model on the priming windows, it is expected to identify the sequence motifs within them and accurately predict the binding affinity of oligo(dT) primers to any given putative priming window.

In our MLP model, one-hot encoding was employed to represent DNA sequences as binary vectors using `sklearn.preprocessing.OneHotEncoder`. Each base is encoded as a unique vector comprising all zeros except for a single one in a designated position: A as (1, 0, 0, 0, 0), T as (0, 1, 0, 0, 0), C as (0, 0, 1, 0, 0), G as (0, 0, 0, 1, 0), and N as (0, 0, 0, 0, 1). Consequently, each priming window of length 30 bp is represented by an array with a length of 150. We initialized a `MLPClassifier` from `sklearn` with an `adam` optimizer by setting all parameters as default. As the extracted "priming windows" might be intergenic instead of the actual priming window (in the minority of cases when the mapped read 1 originate from polyA tail priming), we filtered out the sequences that do not contain an A-SNR of length at least 6 with at most one mismatch.

We trained and evaluated the MLP using the training and test split discussed in Section 2.1. In each training dataset, 1,000 samples were held out as a validation set, and the remaining were utilized for training. In particular, we trained the filtered priming window sequences from the training datasets in batches using `partial_fit` because the training sets were too large to load into memory at once. We evaluated the performance of the trained MLP by calculating the prediction accuracy of the trained MLP on the training datasets, hold-out evaluation sets taken from the training datasets, and the two test datasets.

Note that the potentially mislabeled sequences in the provided training data might cause the performance of the trained MLP to vary across datasets. As discussed in Section 2.5, there are two sets of mislabeled sequences in our training data. The first set contains the background sequences that have the potential to be primed but were not selected for priming (false negatives). The second set contains the intergenic sequences extracted from the biological read 1s that are partially arising from polyA tails (false positives), because we extracted the upstream sequence of biological read 1s from the *genome*. Despite these challenges, our results (Appendix Table 2) indicate that, even though the mean prediction accuracy score of datasets varies (range from 0.72 to 0.92), the trained MLP consistently predicts well across the training set, hold-out set, and test datasets, with a mean accuracy of

[§] https://github.com/pysam-developers/pysam

0.83, 0.84, and 0.89, respectively. We emphasize that the training and test datasets were randomly selected at the beginning of our pipeline and were used across the work (Section 2.1). Although the mean accuracy of the test datasets is slightly higher than that of training, the per-dataset accuracy scores are not the highest scores of the selected datasets and are close to most of the other datasets. We also note that the few datasets exhibited relatively lower accuracy, variations in the MLP's performance across different datasets can be expected due to imperfections in the labeling process(Section A.4).

Table 2. The per-dataset accuracy scores of the MLP predictions on the training sets (the *Training* column), the corresponding hold-out sets (the *Hold-out* column), and the two independent test sets (the *Test* column) as in Appendix Table 1. The last row with the name *Mean* represents the mean accuracy score of each column. The training and hold-out sets in each row are from the same training dataset. The *Test* column shows the accuracy scores of the two test datasets independent of the training datasets (Section 2.1).

#	Training	Hold-out	Test
1	0.84	0.85	
2	0.92	0.92	
3	0.88	0.90	
4	0.89	0.89	
5	0.73	0.72	
6	0.82	0.83	
7	0.87	0.86	
8	0.72	0.72	
9			0.90
10			0.88
Mean	0.83	0.84	0.89

A.6 Details of data simulation

The simulated datasets were generated according to a real-world 10X 1k PBMC scRNA-seq sample download from 10x Genomics website ¶. We first processed the scRNA-seq datasets using `simpleaf` (version 0.16.0)(He and Patro, 2023) with the human *spliceu* reference (Section 2.2) to generate the spliced, unspliced, and ambiguous count matrices. Next, these count matrices were loaded into Python as an `AnnData` object (Virshup et al., 2021) using `pyroe` (He and Patro, 2023). The ratio of spliced to unspliced counts of each gene in each cell was computed by dividing the spliced count matrix by the unspliced matrix element-wisely. For each gene in each cell (gene, cell pair), the total number of reads we expected to simulate was equal to its ambiguous count. The expected number of reads simulated from spliced and unspliced transcripts was decided by the ratio of its spliced to unspliced count. The initial Phred scores for simulated, error-free reads were set at 37. To note that because we allow at most $100,000$ attempts when simulating each read, the final number of simulated reads might be different from the expected number of reads (Section 2.8).

To replicate realistic Illumina errors in the simulated reads, we utilized `InSilicoSeq` (`ISS`) (Gourlé et al., 2019) to introduce errors into our simulated reads. We first developed a custom error model using `ISS` with the default settings, which requires paired-end alignments from a real dataset and the MD SAM tag. Therefore, we aligned the paired-end reads from the 10X 1k PBMC sample using `STAR` and specified the MD tag as an attribute in the output. Next, we passed this error model and the simulated error-free paired-end reads to `ISSS`. `ISS` estimated the per-base quality score for each sequencing base from the 10X 1k PBMC sample, thereby introducing substitution errors into the simulated error-free paired-end reads based on these estimated scores. The paired-end reads with errors introduced were exported into two separate `FASTQ` files using `SeqIO` from `Biopython`. We then aligned the simulated read 2s using `STAR` against the *spliceu* index using similar flags as discussed in section A.3. Notice that although we simulated pair-end reads, we aligned only their read 2, rather than the paired-end reads because the true splicing status of simulated reads was already known when simulating the reads — reads are given rise to the selected transcripts with a known splicing status.

Appendix B Additional details of `Forseti` evaluation

In Section 3.2, we discussed the overall performance of `Forseti` on the four evaluation read sets, two from simulation and two from the experimental test datasets. Here, we break down each evaluation read set into two groups, spliced and unspliced reads, and discuss the performance of `Forseti` on each group under two circumstances, ambiguous prediction excluded or included.

In Appendix Table 3, we show the number of spliced and unspliced reads in each evaluation set. In the two simulated evaluation sets, the number of unspliced reads exceeds the number of spliced reads. However, in the two experimental evaluation sets, spliced reads take up the majority. We emphasize that splicing-aware read simulation is an unsolved research question (Yan et al., 2023) and the discrepancy in the number of spliced and unspliced reads in the simulated and experimental evaluation sets comes from two factors. First, the two simulated sets are based on the UMI count, instead of read counts, of a real scRNA-seq dataset. As shown in He et al. (2023), when using *spliceu* references, there are usually more unspliced UMIs than spliced in scRNA-seq. Secondly, we applied an "accept or skip" strategy to simulate reads (Section 2.8). Specifically, when generating a fragment, we allow at most a certain number of attempts. If we exceed the number of maximum attempts but still haven't generated a read with the expected class, we skip the generation of this fragment and move to the next one. The reason we chose this strategy is that

¶ https://www.10xgenomics.com/datasets/1-k-pbm-cs-from-a-healthy-donor-v-3-chemistry-3-standard-3-0-0

spliced transcripts usually contain fewer polyA stretches than unspliced transcripts, so the generation of spliced fragments is harder (sometimes impossible) than unspliced fragments, especially when we require read 2 must be contained within an exon and read 1 must not be contained within the same exon. In other words, our simulation strategy is positioned to generate more unspliced fragments than spliced ones because of the intrinsic constraints placed on the generating procedure. Furthermore, although we saw a class imbalance in the experimental evaluation sets, we still utilized all possible reads to evaluate the performance of `Forseti` instead of manually balancing the number of reads in the spliced and unspliced classes, because identifying the splicing status of reads is a challenging and ongoing research task, we would not like to waste any reads that can be used to evaluate the performance of our model, and we expect such an intrinsic class imbalance arises as a natural and fundamental characteristic of the problem we are attempting to address.

Table 3. Consolidated table showing the number of spliced and unspliced reads, and the true positive rate (TPR) and false positive rate(FPR) values of `Forseti` predictions of the evaluation sets, two from simulation and two from experimental scRNA-seq datasets. Ambiguous predictions were excluded when computing the TPRs and FPRs. This table provides the underlying data for fig. 3. The *Experiment* column indicates the name of the evaluation sets. FA stands for fundamental ambiguity. The two experimental sets are named by their source datasets, GSE122357 and GSE125188. The columns named *Spliced*, *Unspliced*, and *Total* represent the true number of spliced and unspliced, and total reads, respectively. The *Definitive prediction* represents the total number of reads assigned a definitive splicing status, either spliced or unspliced.

#	Experiment	Spliced	Unspliced	Total	Definitive prediction	Spliced		Unspliced	
						TPR	FPR	TPR	FPR
1	simulation with FA	2,532,009	3,758,706	6,290,715	1,510,121	0.91	0.38	0.62	0.09
2	simulation without FA	852,206	1,620,437	2,472,643	2,090,408	0.94	0.38	0.62	0.06
3	GSE122357	163,133	19,853	182,986	142,420	0.91	0.19	0.81	0.09
4	GSE125188	845,724	160,593	1,006,317	800,405	0.90	0.29	0.71	0.10

Appendix Table 3 also shows the true positive rate (TPR) and false negative rate (FPR) for spliced and unspliced reads in each evaluation set. As explained in Section 2.8, read-level `Forseti`, the model we proposed in this work, currently lacks the ability to predict the splicing status of fundamental ambiguous reads — reads with their entire corresponding cDNA fragment arising from an exon, because the spliced and unspliced version of each transcript share exons and the local context of the fragments under both statuses are, therefore, equivalent. Therefore, when we calculated the FPR and TPR, and the area under the ROC curve (AUC) of `Forseti` predictions on the evaluation sets (Appendix Table 3 and fig. 3), we excluded ambiguous predictions. An alternative strategy could be to set aside fundamentally ambiguous reads as their own class, and perform three-class prediction. This may ultimately be a more meaningful classification strategy. Nonetheless, in the current work, we retained the simple two-class classification. In Appendix Table 3, the consistently high TPRs for spliced predictions across all evaluation sets suggest that `Forseti` has a high sensitivity for spliced reads, while the relatively higher false positive rate for the two simulated sets than the experimental sets also suggest that, because we ignored the correlation between spliced and unspliced transcripts, the simulated spliced reads are usually harder to predict than in the real cases. On the contrary, for unspliced reads, the consistently low FPRs suggest that `Forseti` has high specificity for unspliced reads. Meanwhile, the relatively low TPR of the simulated sets than that of the experimental sets also suggests that, sometimes it is conservative to assign unspliced status to reads and assigns ambiguous instead. One reason might be that, in addition to the true transcript and priming window that was used to simulate a read, there might exist other priming windows that, when paired with the simulated read, can result in better prediction scores because of better-defined polyA and fragment length. If those better priming windows appear on both spliced and unspliced transcripts, i.e., on exons or polyA tails, then `Forseti` will assign an ambiguous status. This challenge is potentially exacerbated in our simulated data because when simulating reads, once we select a reference, we repeatedly, in up to $100,000$ trials, simulate qualified reads from all potential priming windows on the selected reference. This strategy might produce reads that do not score the highest by `Forseti` when pairing with their true origin compared with other potential priming windows. Nonetheless, even with all these imperfections, the overall performance of `Forseti` is still good, supported by the consistently high AUC scores in Figure 3, and it performs much better than existing naïve strategies widely used to assign splicing status.

One advantage of being a probabilistic model is that, for those fundamentally ambiguous reads that our model is currently unable to handle, their splicing status will remain ambiguous in `Forseti`'s prediction, with a probability of being spliced as exactly 0.5. In the two experimental datasets, reads that remain with an ambiguous splicing status after `Forseti` account for 14% and 20%, respectively, suggesting that fundamental ambiguity is cell-type specific. In the simulated data with fundamental ambiguity, ambiguous reads account for $\sim 71\%$ of reads, mainly because our simulation setting prefers generating fundamentally ambiguous reads (Section 2.8). In the simulated dataset without fundamental ambiguity, this percent becomes 15%, consistent with the experimental sets. This suggests that our simulation that allows fundamental ambiguity is unrealistic (i.e. is overly pessimistic) and that the degree of such ambiguity is likely substantially lower in real-world, experimental data.

Although considering those ambiguous predictions when evaluating the performance of `Forseti` is not reasonable, because our model would not give a prediction to those reads and they stayed in the ambiguous status as in the original input, in the following text, we still discuss the metrics explained in Section 3.2 and above paragraphs with ambiguous predictions included. As shown in Appendix Figure 1, for simulation with fundamental ambiguity, this rather unrealistic simulation is dominated by fundamentally ambiguous reads ($4,775,594$ out of $6,290,715$ or $\sim 71\%$), when including ambiguous predictions, the AUC score dropped from 0.92 to 0.68. However, it is still much higher than the 0.5s of the baseline models. Similar drops for the TPR and FPR when included ambiguous predictions were observed in Appendix Table 4 as well, because the ambiguous predictions were treated as false predictions. Apart from this overly pessimistic case, the TPR, FPR, and AUC of other evaluation sets remain relatively good,

as shown in Appendix Figure 1 and Appendix Table 4. We note that the middle, thin part of the curves in Appendix Figure 1 is caused by ambiguous predictions. Because `Forseti` assigned a probability of 0.5 to all ambiguous predictions, a tiny increment of the threshold parameter of ROC from 0.5 is accompanied by a drastic change of TPR and FPR, causing a thin line in the middle of the ROC curves without any supporting data point.

Table 4. This table shows the true positive rate (TPR) and false positive rate (FPR) values of `Forseti` predictions on the four evaluation sets as in Appendix Table 3, but ambiguous predictions were included when calculating TPR and FPR.

#	Experiment	Total	Definitive prediction	Spliced		Unspliced	
				TPR	FPR	TPR	FPR
1	simulation with FA	6, 290, 715	1, 510, 121	0.38	0.05	0.08	0.04
2	simulation without FA	2, 472, 643	2, 090, 408	0.80	0.32	0.52	0.05
3	GSE122357	182, 986	142, 420	0.78	0.17	0.71	0.08
4	GSE125188	1, 006, 317	800, 405	0.72	0.24	0.60	0.08

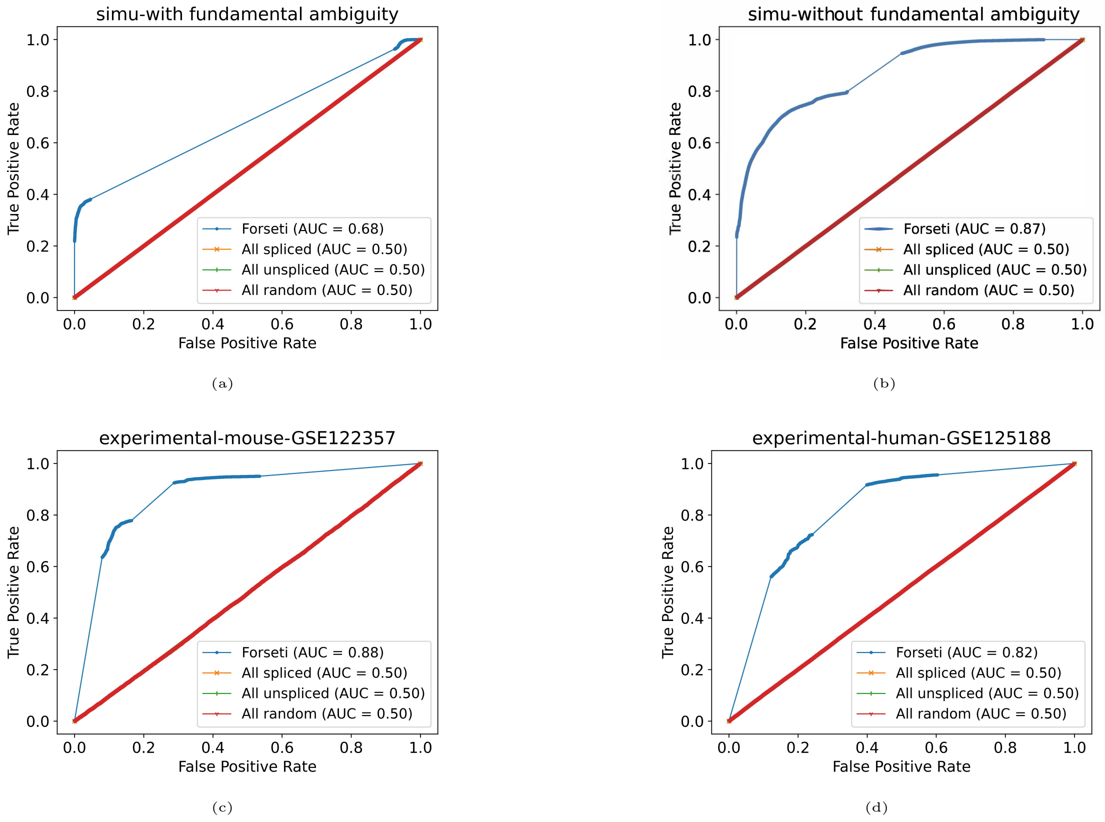

Appendix Figure 1. The ROC curves that are analogous to those in Figure 3, but reads predicted as ambiguous were considered as false predictions and included when calculating the TPR and FPR).
