# Supplementary figures and images for: Forseti: a mechanistic and predictive model of the splicing status of scRNA-seq reads
Source: Bioinformatics. 2024 Jun 28;40(Suppl 1):i297–306. doi: 10.1093/bioinformatics/btae207 (PMC11256924; doi:10.1093/bioinformatics/btae207)

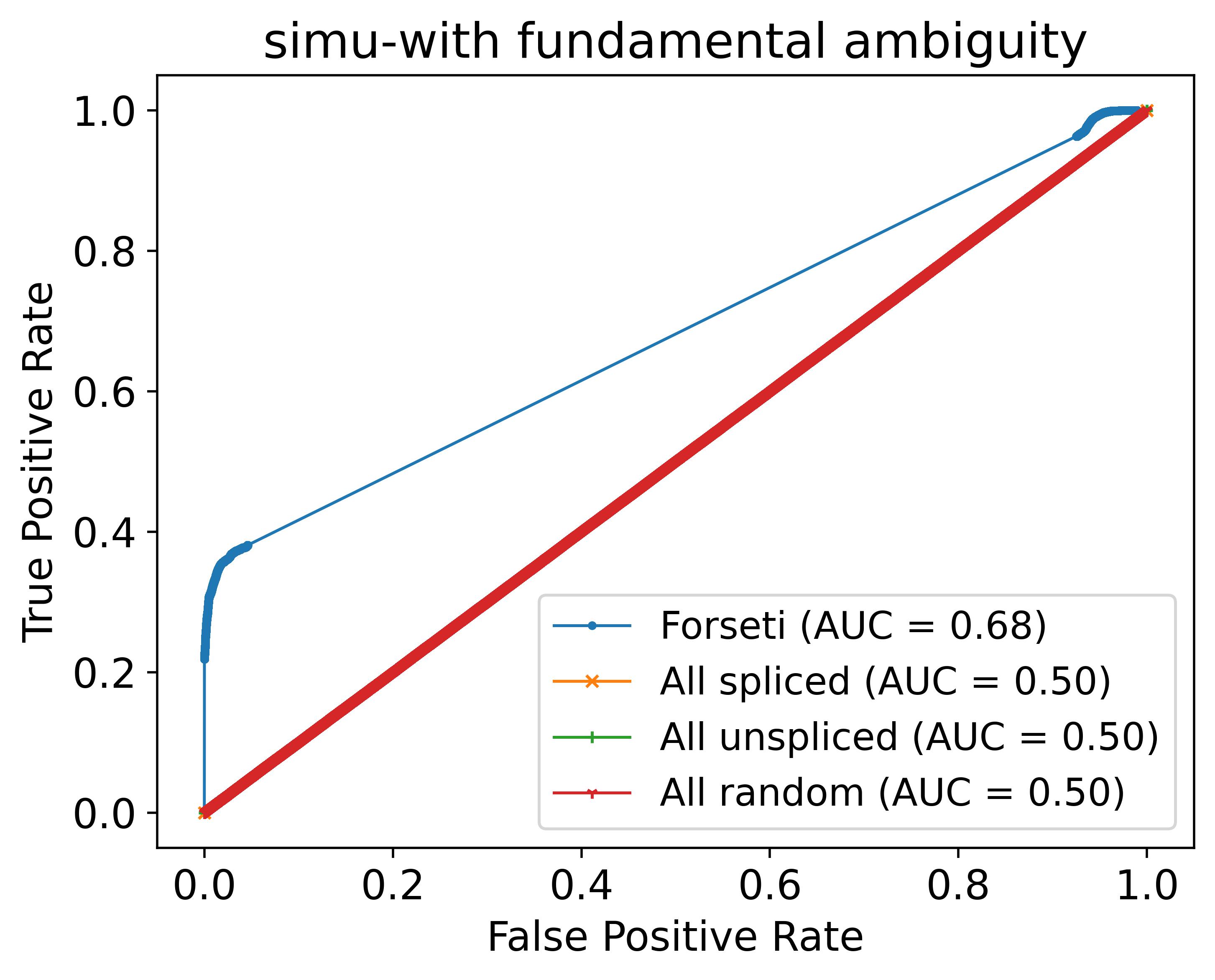

Supplement: btae207_Supplementary_Data [file btae207_supplementary_data.zip › btae207_Supplementary_Data/Patro.258.supp.2.jpg]

simu-with fundamental ambiguity

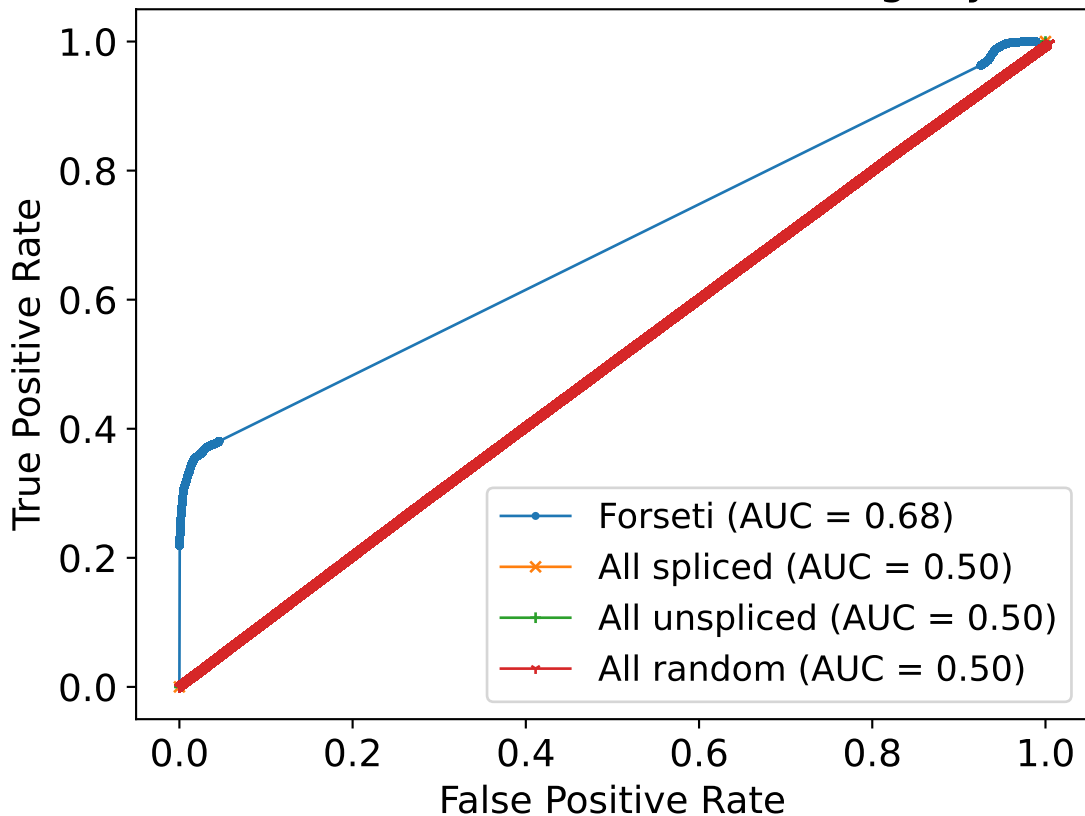

Supplement: btae207_Supplementary_Data [file btae207_supplementary_data.zip › btae207_Supplementary_Data/Patro.258.supp.2.pdf]

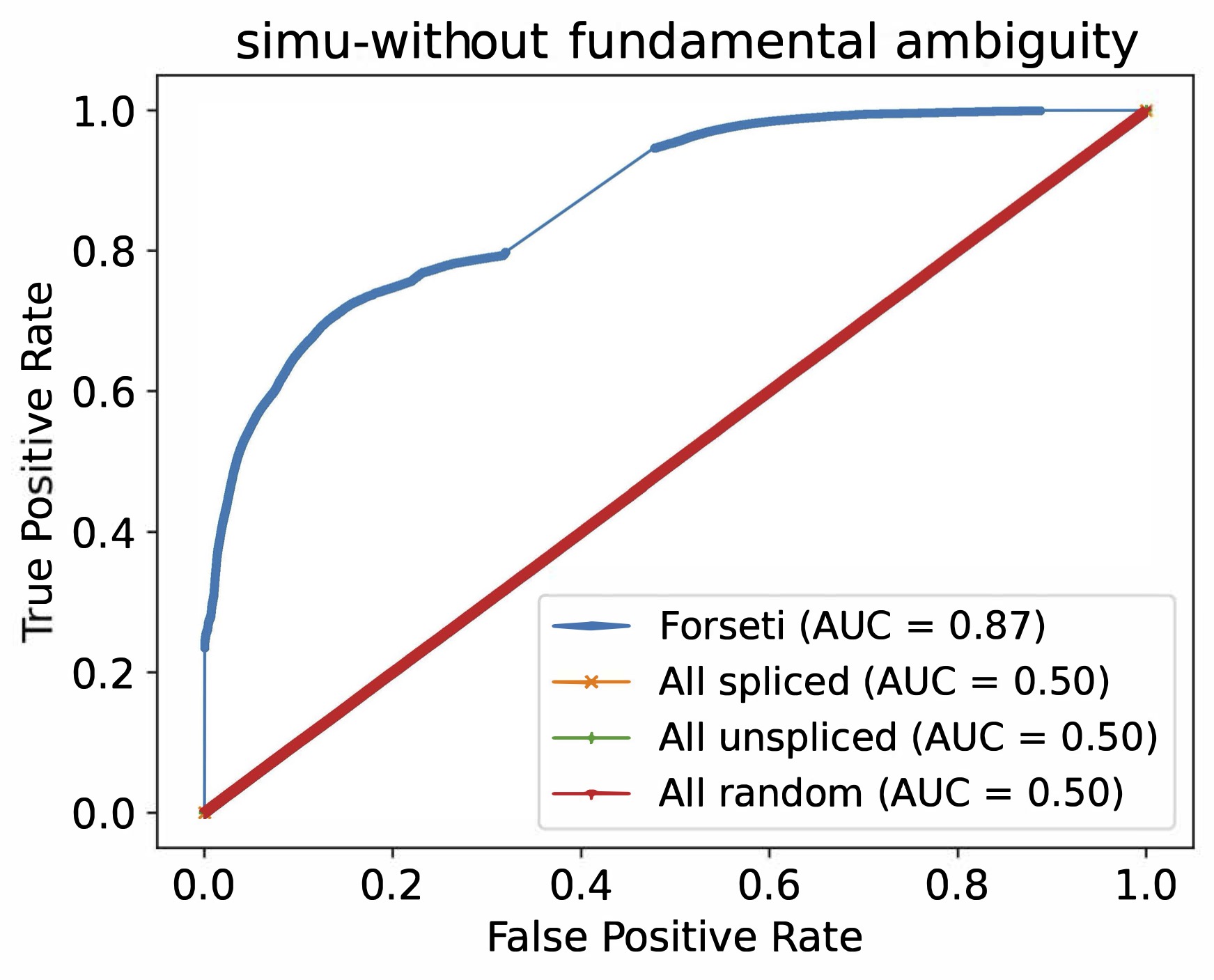

Supplement: btae207_Supplementary_Data [file btae207_supplementary_data.zip › btae207_Supplementary_Data/Patro.258.supp.3.jpg]

simu-with fundamental ambiguity

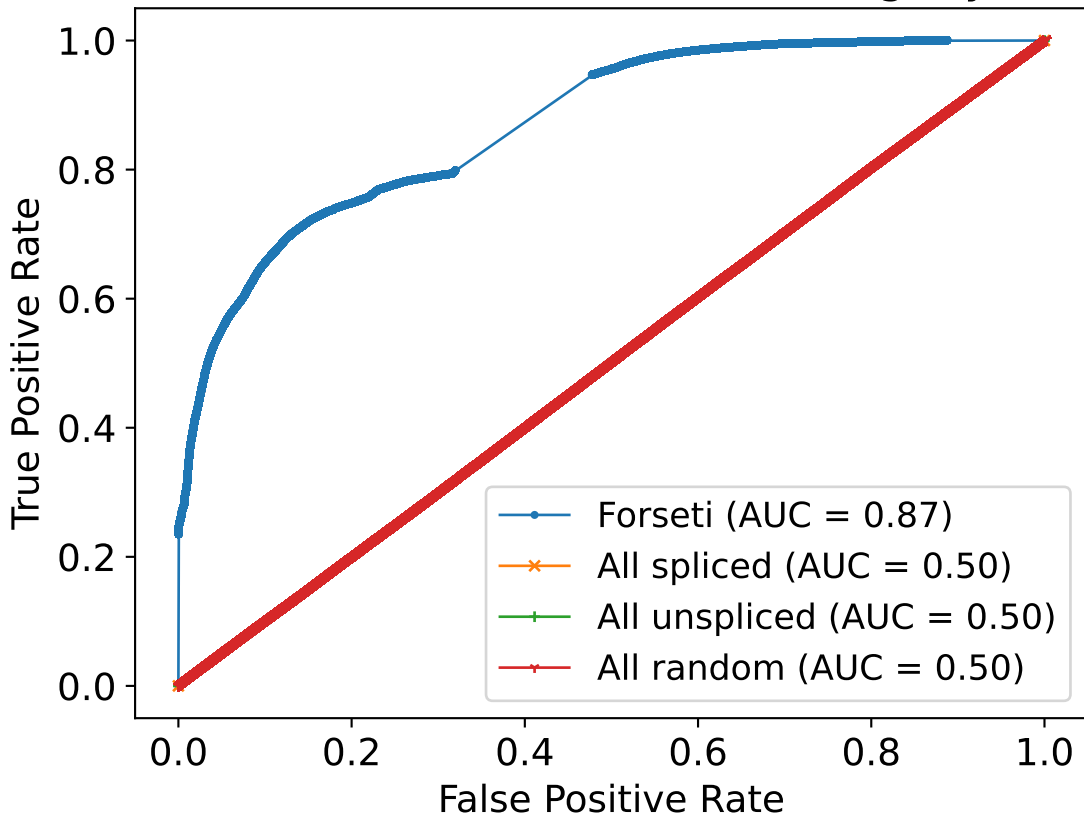

Supplement: btae207_Supplementary_Data [file btae207_supplementary_data.zip › btae207_Supplementary_Data/Patro.258.supp.3.pdf]

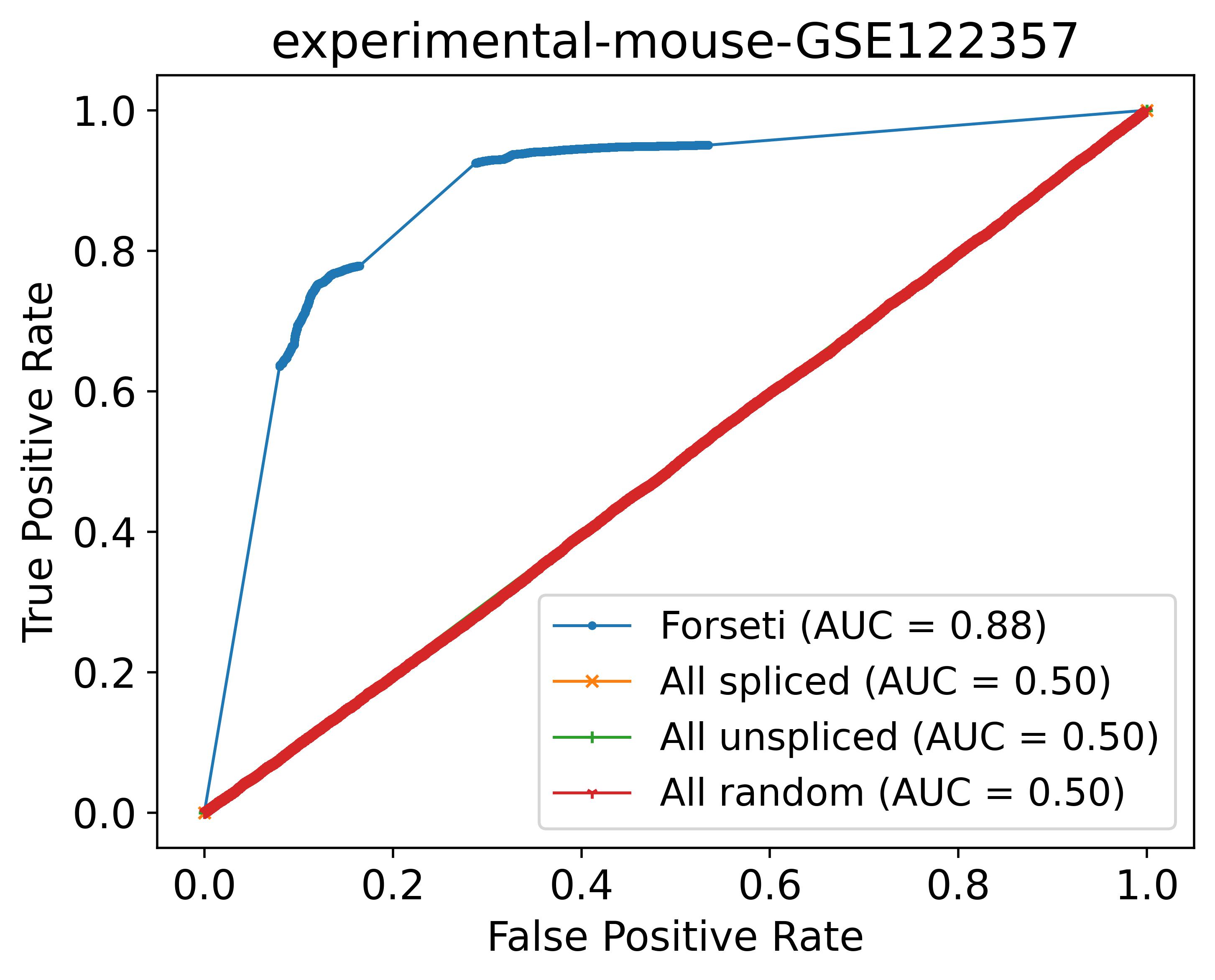

Supplement: btae207_Supplementary_Data [file btae207_supplementary_data.zip › btae207_Supplementary_Data/Patro.258.supp.4.jpg]

experimental-mouse-GSE122357

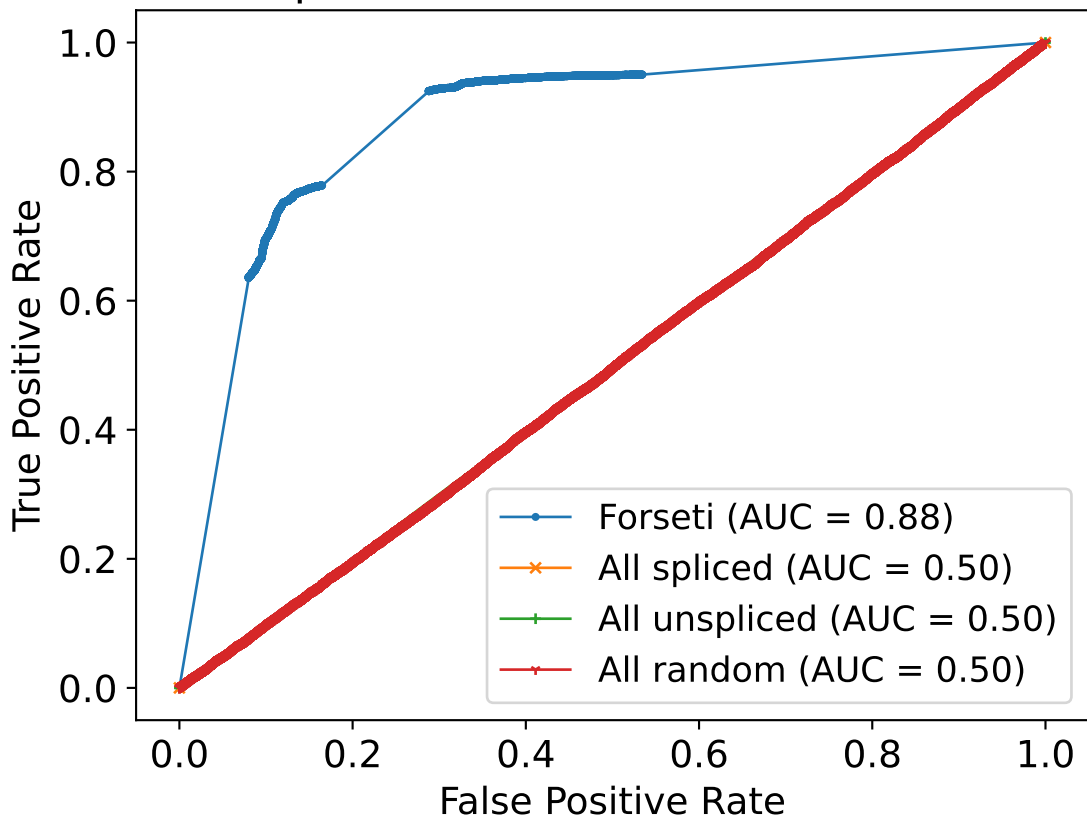

Supplement: btae207_Supplementary_Data [file btae207_supplementary_data.zip › btae207_Supplementary_Data/Patro.258.supp.4.pdf]

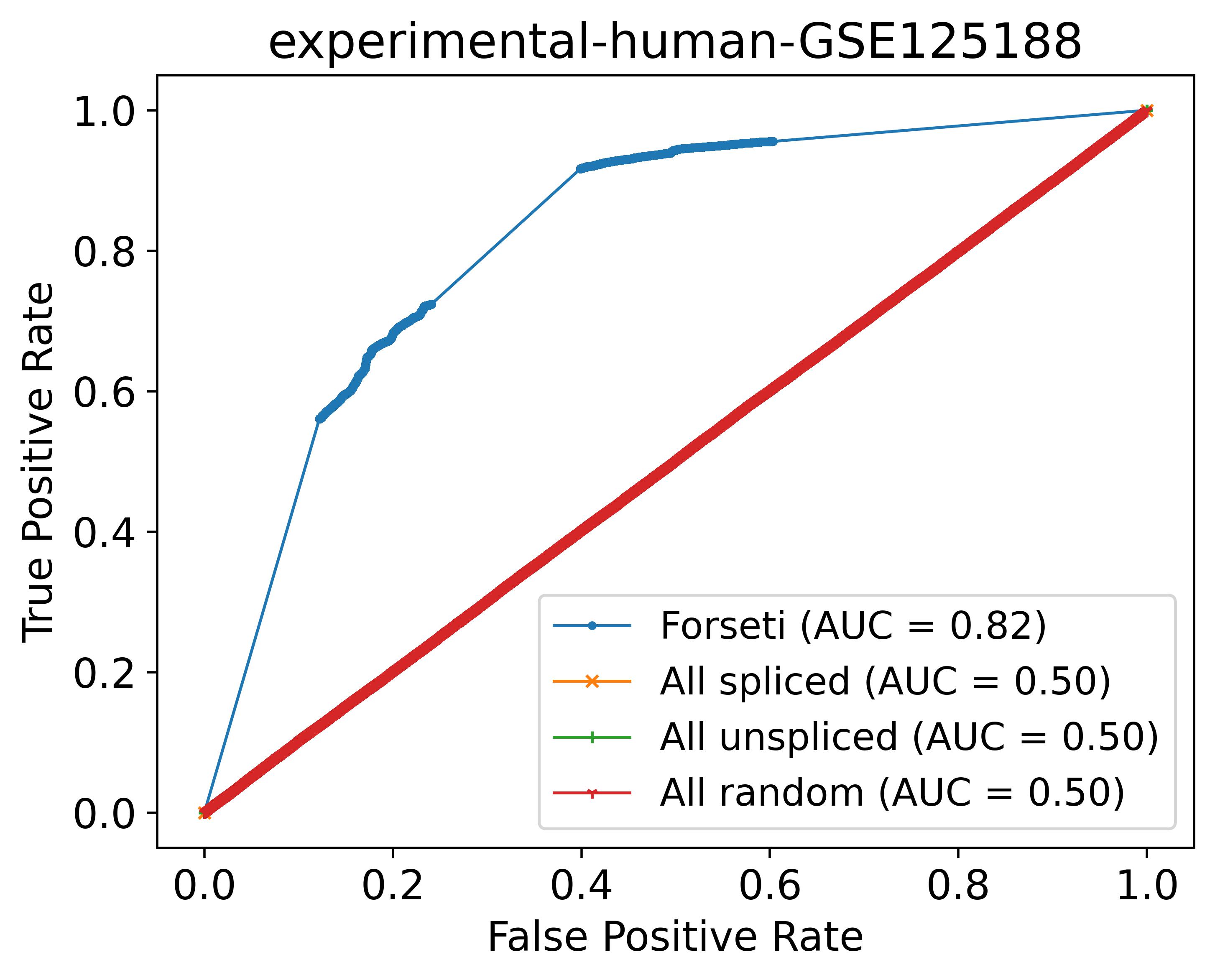

Supplement: btae207_Supplementary_Data [file btae207_supplementary_data.zip › btae207_Supplementary_Data/Patro.258.supp.5.jpg]

# experimental-human-GSE125188

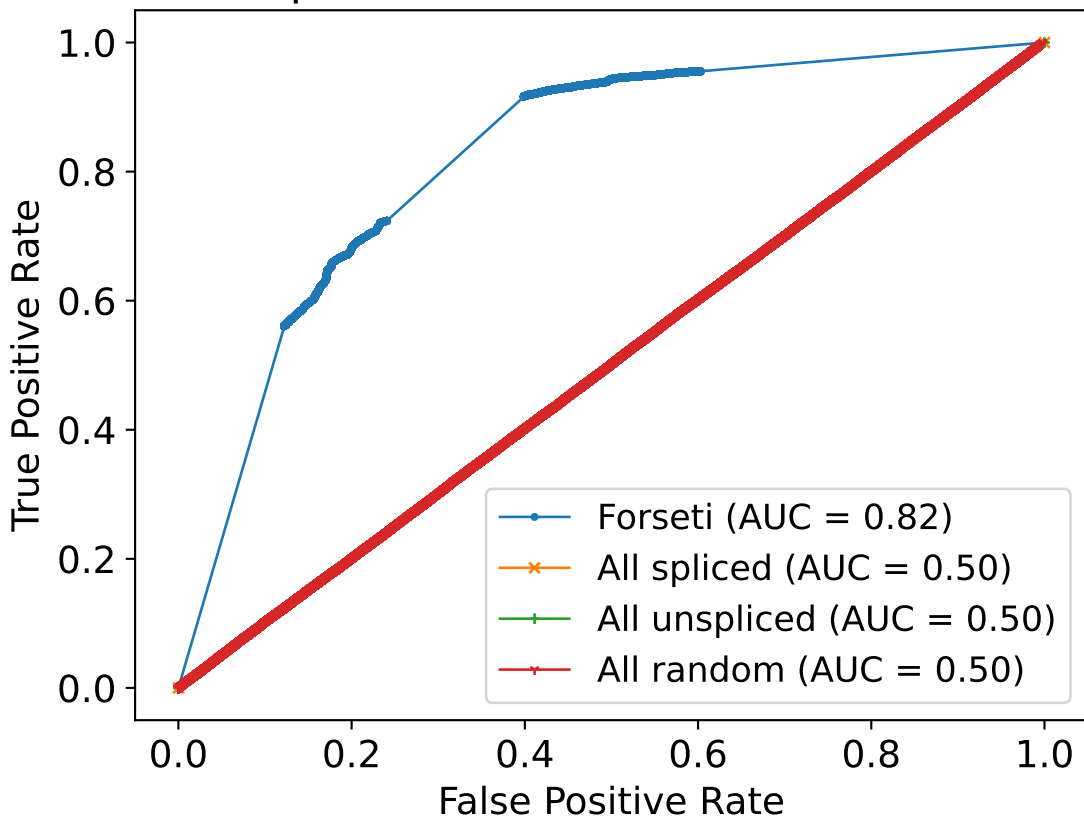

Supplement: btae207_Supplementary_Data [file btae207_supplementary_data.zip › btae207_Supplementary_Data/Patro.258.supp.5.pdf]
